# Sciatica Relieved by Injection

**Published:** 1886-02

**Authors:** 


### SCIATICA RELIEVED BY A SINGLE COCAINE INJECTION.

Dr. W. B. Menz, of Vidalia. La., writes that he was called to a lady, fifty-five years of age, who had been a constant sufferer from sciatica for ten years. The pain was very severe, and extended along the entire length of the nerve. She had run the whole gamut of anti-neuralgic remedies, and had never obtained anything more than very transitory relief. Having with him a vial of a four-per cent. solution of cocaine hydrochloric, Dr. Menz determined to try the efficacy of a subcutaneous injection. The hypodermic needle was inserted deeply over the sciatic foramen, and about twenty drops of the solution were passed into the tissues. The pain ceased almost immediately and during the six weeks that have since elapsed has not returned, although there has been no further treatment, and one injection only was practiced. The relief given by other remedies has never been of more than from two to four hours' duration —*Medical Record.*

